# Morphology and dimensions of the dentogingival unit in the altered passive eruption

**DOI:** 10.4317/medoral.18044

**Published:** 2012-05-01

**Authors:** Francisco Alpiste-Illueca

**Affiliations:** 1 Professor of Periodontics, Valencia University Medical and Dental School. Valencia, Spain; 2 ……..; 3 ….

## Abstract

Objetives: This study define altered passive eruption (APE) and evaluate the morphology of the dentogingival unit. Material and Methods: 123 individuals subjected to clinical examination and parallel profile radiography of the upper central incisor. An evaluation was made of the correlation between the clinical diagnosis of APE and the degree of gingival overlap; by using a 19% overlap to define APE (Kappa concordance index = 0.7). The Mann-Whitney / Wilcoxon test was used to identify the variables influencing APE. Results: Statistically significant differences were observed between the teeth with and without APE: gingival width (p = 0.0073), clinical crown length (p = 0.0000), smiling exposed gums (p = 0.0000), bone crest thickness (p = 0.0030), connective tissue attachment thickness (p = 0.0003) and biological width (p = 0.0015). Conclusions: The APE is characterized by: a gingival overlapping of over 19% of the length of the anatomical crown, increased gingival width and gingival smile; furthermore is associated to a thick bone crest and connective tissue attachment. Statistical analysis confirms two morphological patterns of APE.

** Key words:**Altered passive eruption (ape), radiographic exploration, dentogingival unit (dgu), gingival thickness, plastic periodontal surgery, surgical crown lengthening.

## Introduction

The dentogingival unit (DGU) has been described as a functional unit composed of the epithelial attachment and connective tissue attachment of the gingiva – both of which afford biological protection (1). Gargiulo et al. used the term “physiological DGU” in application to the anatomical complex formed by the gingival margin, sulcus, junctional epithelium and connective tissue attachment, and stressed the importance of the epithelial and connective attachments as the main components of this functional unit (2). These authors described the relationships and dimensions of the DGU components in humans, and their results show the epithelial attachment to measure 0.97 mm on average, and the connective tissue attachment 1.07 mm. The term “biological width” was introduced to describe the space occupied over the tooth surface by the connective and epithelial attachments, this parameter being equivalent to the distance between the bottom of the gingival sulcus and the alveolar bone crest. In humans this distance is 2.04 mm on average (2-4).

The DGU is habitually located close to the cementoenamel junction, the gingival margin slightly covering the limits of the dental crown (5). Different physiological situations do not exhibit this morphological disposition, however, and the gingival margin tends to occupy a much more incisal position – thus giving rise to short clinical crowns. This variation in habitual morphology involving a more coronal periodontium has been referred to as altered passive eruption (APE) or delayed passive eruption (6).

The term “altered passive eruption” refers to the supposed mechanism underlying production of this morphological variant. Tooth eruption comprises two phases (7): an active eruption phase which causes the tooth to emerge into the oral cavity, and a passive eruption phase involving apical migration of the soft tissues covering the crown of the tooth. From the current perspective, the active phase of eruption is defined by emerging motion of the tooth in the occlusal direction until the tooth reaches the occlusal plane of its antagonist. This vertical motion causes the gums to displace along with the crown. With the passive eruption phase, the gums migrate in the apical direction, with gradual exposure of the crown of the tooth and final stable localization of the DGU at cervical level.

However, even if this pathogenic hypothesis of APE were accepted, the literature fails to clarify the circumstances causing arrested tooth eruption and conditioning DGU morphology. Many authors have investigated the causes and mechanisms that may lead to tooth eruption failure, though few studies have related such mechanisms to the morphology adopted by the coronal periodontium (8).

The purpose of the study was to further knowledge of the morphological features of APE at DGU level, with the following specific aims: (a) Definition of APE based on gingival overlap on the anatomical crown; (b) The determination of possible differences at DGU level between teeth with and without APE; and (c) Confirmation of the existence of different morphological patterns of APE.

## Material and Methods

A total of 123 individuals participated in the study. The selection focused on ensuring the maximum inclusion of subjects with upper anterior teeth presenting clinical evidence of APE. Five inclusion criteria were defined: age between 20 and 40 years, the absence of upper anterior tooth restorations, high standard of oral hygiene and gingival health (no bleeding on probing and no plaque in the examination area), the absence of previous orthodontic correction, and occlusal attrition index of Smith and Knight ≤ 2. The protocol of the study was accepted by the Committee on Ethics in Human Research of the University of Valencia and written informed consent was granted from all subjects.

Two types of exploration were carried out in second sextant: clinical, and one parallel profile radiography (PPRx) on the maxillary left central incisor (tooth 21). The same examiner (F.A.) performed all clinical and radiography (PPRx) recording.

Clinical exploration

**Figure 1:**
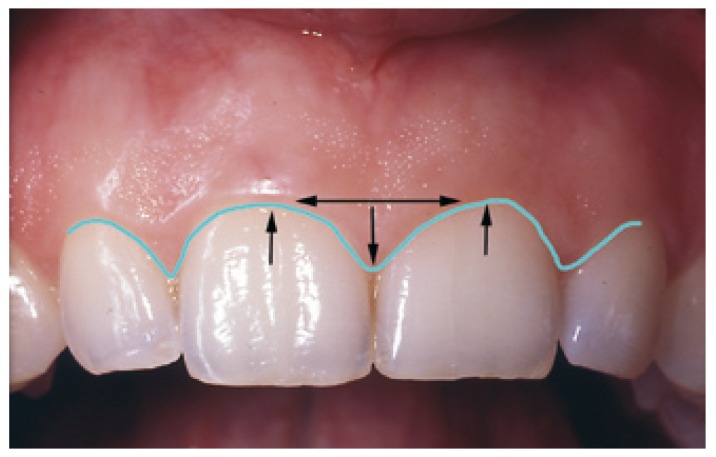
Clinical diagnostic criteria of altered passive eruption (APE).

Two subjective criteria were contemplated for the clinical diagnosis of APE: (a) excessively flattened gingival festooning, and (b) a disproportionate papilla base width in relation to the height reached by the tip. The APE was diagnosed when these criteria were met in the context of a patient with a clinically apparent short dental crown (Fig. 1).

The molar and canine Angle occlusion class was recorded. Upper lip height was measured as the distance from the base of the nose to the lower margin of the lip, with an evaluation of the amount of gingival tissue exposed over the four upper incisors on smiling.

Probe depth (in mm) was evaluated at three points on the vestibular aspect of each of the sextant teeth. The keratinized gingival width was measured on each tooth. The mucogingival line was located by means of the Coppes technique (pushes of the mucosa), while the gingival width was determined using a graded periodontal probe, measuring from the mucogingival line to the gingival margin in the medial zone and expressing the results in millimeters. And the length of the clinical crown was measured. Finally, occlusal attrition of the teeth was determined applying the index of Smith and Knight (9) with modification for this study: 0 = intact incisal edge; 1 = non-visualization of the enamel lobes; 2 = the dentin is seen by transparency; 3 = exposed dentin.

Parallel Profile Radiography (tooth 21)

**Figure 2:**
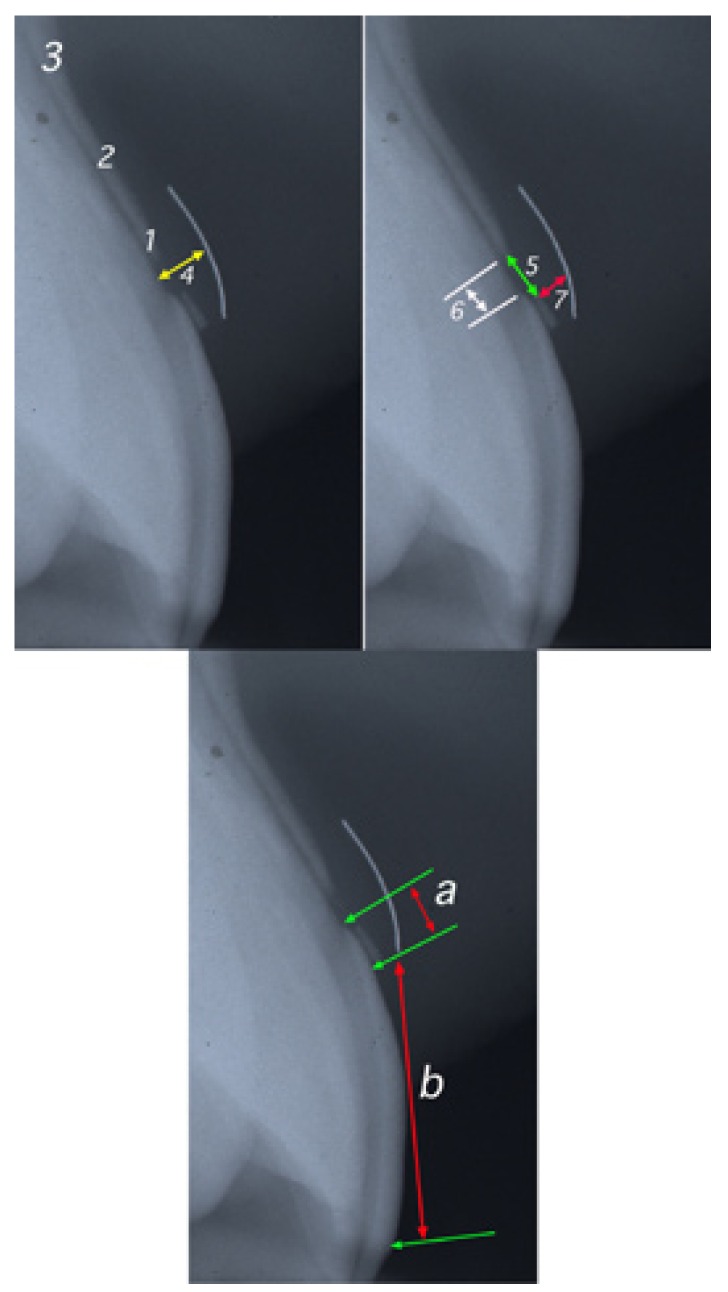
PPRx. 1, 2 and 3 thickness of the bony table, 4 thickness of the connective tissue attachment, 5 biological width, 6 the distance from the cementoenamel junction to the crest, 7 the thickness of the free gingiva. a- discrepancy: amount of mm. that encía covers the anatomical crown, b- crowns clinic, a + b = crowns anatomical of the tooth.

The purpose of this study was to determine the dimensions of the DGU components for tooth 21 (Fig. 2). Parallel profile radiography (PPRx) was used to this effect (4). Measurements were obtained of the thickness of the buccal bone plate at crest level, at the middle third, and at the apical third. The thickness of the connective tissue attachment was determined at the cementoenamel junction and at middle-third and crest level. Measurements were also obtained of the biological width, the distance from the cementoenamel junction to the crest, the thickness of the free gingival at the base and middle-third, discrepancy, and gingival overlap (4).

The reproducibility of the PPRx technique was assessed by means of a double experiment. In order to evaluate observer error, 40 radiographs were randomly selected from the original sample, and double measurements were made over these radiographs on separate days (total: 560 measurements). The precision of the technique was then evaluated by performing the radiographs in duplicate - each time completely dismounting and again mounting all the pieces of the paralleling system (total: 840 measurements).

The Pearson coefficient r (linear correlation) was estimated, and linear regression analysis was performed, yielding estimations of the constant (a) and slope (b) of the regression line, with estimation of the corresponding 95% confidence interval (95% CI). The double measurements of the 40 radiographs showed that method reproducibility could be accepted at a 95% confidence interval for all the variables. In the second experiment, all measurements satisfied the conditions required to ensure reproducibility, except the variable “biological width”, which failed to coincide on a pair of radiographs, and the variable “distance between the cementoenamel junction and bone crest”, which failed to coincide on two pairs of radiographs.

Study design and statistical analysis

The variable discrepancy is obtained from the PPRx study and reflects the discrepancy between the length of the clinical crown and the length of the anatomical crown - being equivalent to the amount (in mm) of gingival tissue covering the enamel surface as determined at the cementoenamel junction. In turn, the variable overlap refers to the percentage anatomical crown covered by the gingival (Fig. 2).

A descriptive statistical analysis was first made of the clinical and radiological variables for all the subjects included in the study. This was in turn followed by an analysis of the correlation between the clinical diagnosis of APE in application to tooth 21 and the variables discrepancy and overlap. After demonstrating this correlation, an evaluation was made of the percentage overlap that best distinguishes (i.e., with the least possible error) between the clinical diagnosis of APE and non-APE tooth status. In this way APE of tooth 21 is defined according to percentage overlap.

The study sample was divided into two groups: teeth 21 with APE and teeth 21 without APE. The purpose of this division was to identify the variables related to APE and the particularities that may help differentiate the condition.

On the other hand, we also attempted to identify the two morphological patterns proposed in the literature (6) in our series of teeth 21 with APE (APE type 1 / APE type 2).

The Mann-Whitney / Wilcoxon test was used throughout the study to demonstrate the relations between the different variables, accepting a 5% significance level in all cases (α = 0.05).

## Results

The study comprised a total of 123 individuals (27 males and 96 females) with a mean age of 28 ± 7.5 years.

Definition of APE

A first analysis was made of the correlation between the clinical diagnosis of tooth teeth 21 (i.e., with or without APE) and the variables discrepancy and overlap, based on the nonparametric Mann-Whitney / Wilcoxon test. The p-value obtained for both contrasts was under 0.0001, thus indicating that the clinical diagnosis is closely related to the overlap and discrepancy values obtained.

**Figure 3:**
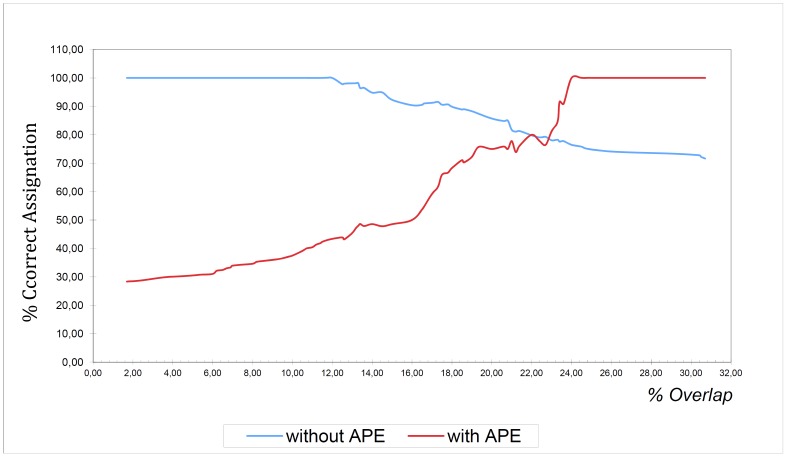
The blue line reflects the proportion of cases not presenting APE for an overlap less than or equal to that indicated on the abscissas axis. The red line indicates the proportion of cases with APE for overlap values greater than or equal to that indicated on the abscissas axis.

An analysis (Fig. 3) was in turn made of the percentage of correct and erroneous clinical diagnoses according to percentage overlap (abscissas); these percentages were calculated based on the hypothesis that an overlap threshold exits beyond which the tooth in question is considered to present APE. Based on the graphic representation, it may be concluded that for a percentage overlap of less than 18%, the tooth in question is well erupted (90% of cases do not present APE), while for percentage values above 24% APE is considered to be present, and in the rest of cases (18-24% overlap) tooth status is doubtful. To the effects of the present study we decided to determine a percentage cutoff regarding the variable overlap capable of discriminating between APE and non-APE tooth status with the least error possible. The value thus determined was 19%, yielding a percentage coincidence with the clinical diagnosis of 87.8% and a Kappa concordance index of 0.7.

Thus, from this perspective, the upper central incisor is considered not to present APE if the percentage overlap is ≤ 19%, while APE status is accepted for an overlap of over 19%. Based on this criterion, APE was observed in 32 of the 123 maxillary left central incisors subjected to PPRx study.

The degree of incisal attrition was found to be low especially in the teeth with APE: 28 presented attrition degree ≤ 1, while only four teeth corresponded to degree 2. Therefore, in our series of teeth, attrition was scantly relevant to crown length.

Characteristics of the teeth 21 with APE

The Mann-Whitney / Wilcoxon test was applied to the clinical variables and PPRx parameters, considering both groups of teeth ( Table 1 and  Table 2).

**Table 1:**
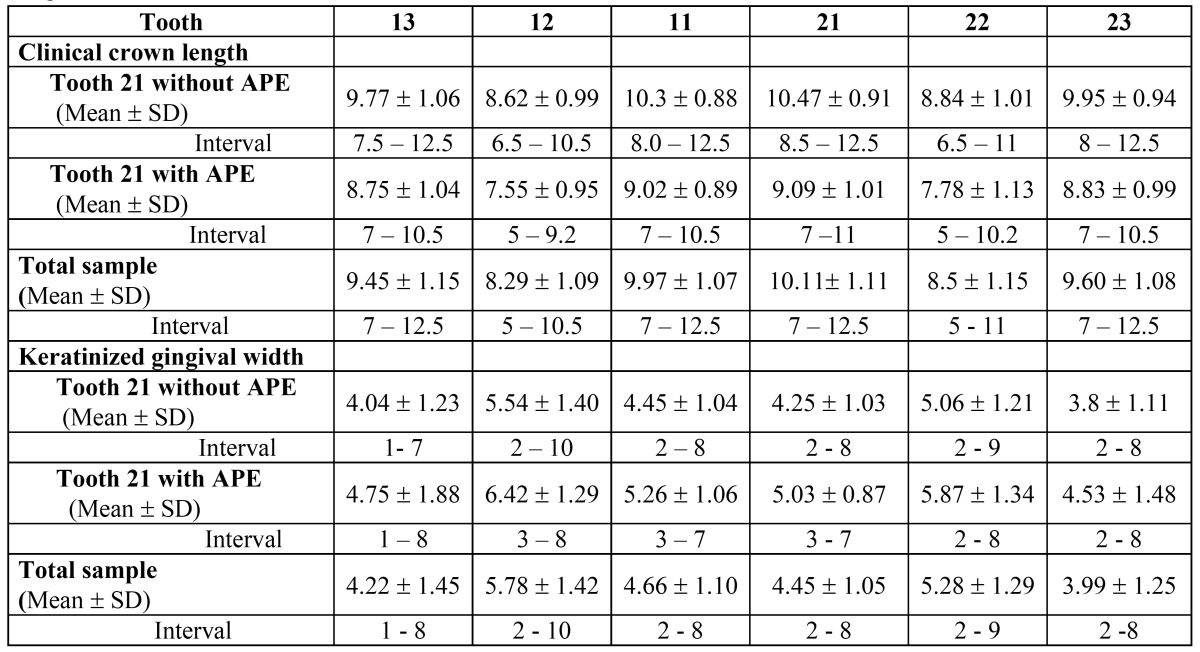
When tooth 21 presented APE (overlap > 19%), the lengths of the clinical crowns of all the teeth in the upper anterior sextant were significantly less than in the rest of the series, while in contrast keratinized gingival width was greater– with the sole exception of the canines.

**Table 2:**
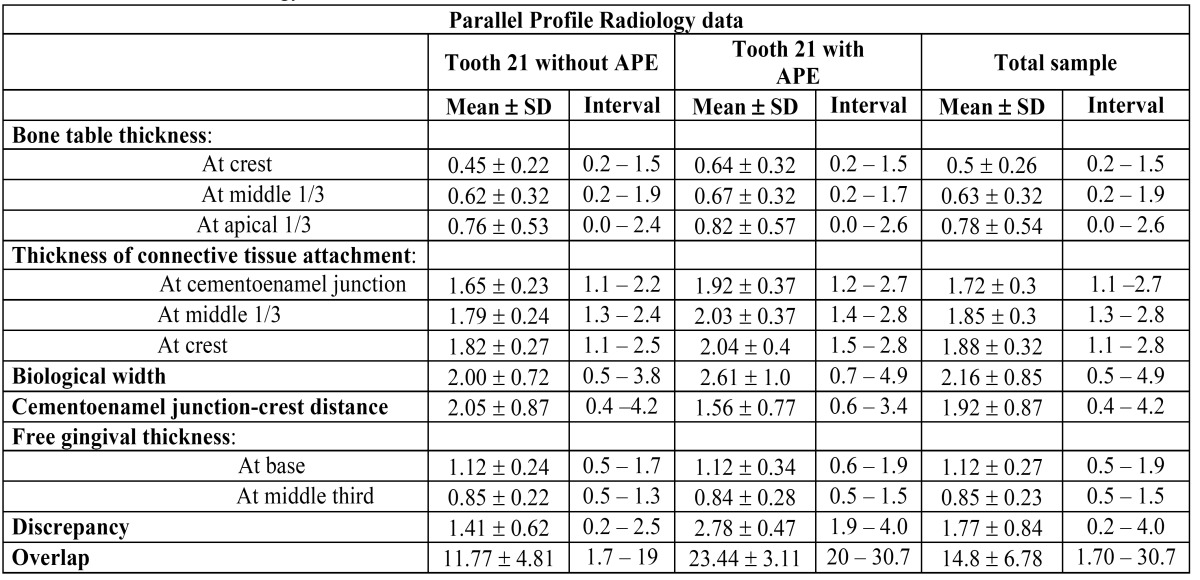
Parallel Profile Radology data.

No significant differences were observed between the presence of tooth 21 with APE and the clinical variables age and sex, Angle molar and canine occlusion class, lip-nose distance and probe depth.

Tooth 21 with APE was seen to expose significantly more gingival tissue on smiling (mean 2.27 ± 1.73 mm) than the tooth 21 without APE (0.88 ± 1.40 mm), (p < 0.0000).

A strongly significant association was observed between tooth 21 with APE status and clinical overbite of teeth 12, 11, 21 and 22 (p = 0.0005, 0.0030, 0.0030 and 0.0009, respectively) – the latter being defined when in maximum intercuspid relation the observed overlap of the crown of the upper incisors over the lower incisors exceeds one-third of the length of their crowns. When tooth 21 presented APE (overlap > 19%), the lengths of the clinical crowns of all the teeth in the upper anterior sextant were significantly less than in the rest of the series (p = 0.0000), while in contrast keratinized gingival width was greater– with the sole exception of the canines [p-value: tooth 13 (0.0744), tooth 12 (0.0012), tooth 11 (0.0051), tooth 21 (0.0073), tooth 22 (0.0005), tooth 23 (0.0877)], ( Table 1).

 Table 2 presents the average dimensions for each variable examined in the PPRx study. Both bone crest thickness (p= 0.0030) and connective tissue attachment thickness [at cementoenamel juntion (p= 0.0003), at middle 1/3 (p = 0.0015), at crest (p= 0.0109)], and biological width length (p= 0.0015), were found to be significantly greater in teeth 21 with APE. In contrast, the distance from the cementoenamel junction to the crest was significantly shorter than in the teeth 21 without APE (p= 0.0036). No significant correlation was recorded between teeth 21 with and without APE in: free gingival thickness, and the thickness of the buccal bone plate at the middle third and at the apical third .

Verification of the morphological patterns in the APE group

An evaluation was made of the possible presence in our series of the two morphological patterns of APE described in the literature: type 1, characterized by a long distance from cementoenamel junction to bone crest together with low discrepancy; and type 2, defined by a short distance from cementoenamel junction to crest together with important discrepancy (6,10). To this effect, a cluster analysis was performed with the 32 teeth 21 presenting APE, dividing the series into two groups (24 teeth in one and 8 in the other) ( Table 3). Both the average distance from cementoenamel junction to bone crest and the mean discrepancy differed significantly between the two groups (p = 0.0001 and p = 0.0032, respectively).

**Table 3:**
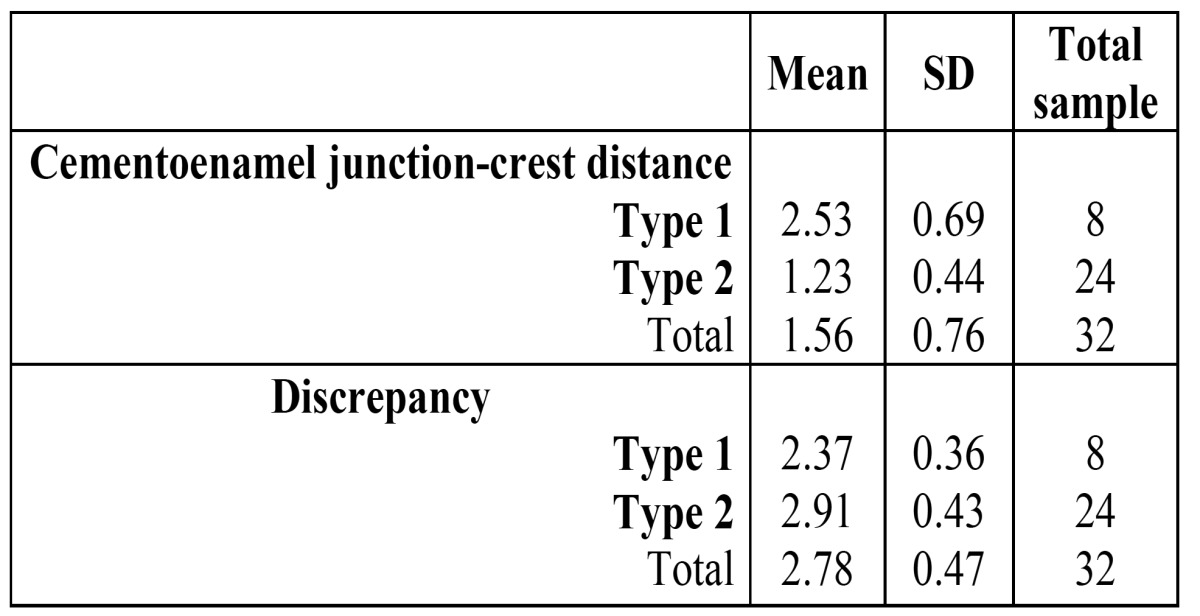
Morphological patterns of APE (Type 1 and Type 2).

## Discussion

The literature lacks references for calibrating the magnitude of gingival overlap characterizing APE. The PPRx allows us to measure discrepancy and the degree of overlap (4). In this way it is possible to correlate the clinical diagnosis with the radiological diagnosis. Accordingly, it was concluded that a gingival overlap equivalent to over 19% of the anatomical crown height is equivalent to the clinical diagnosis of APE (Kappa concordance index 0.7).

Some authors indicate that gingival sulcus probe depth is important for the diagnosis of APE, and claim that depths of over 3 mm without concomitant pathological signs are suggestive of APE (10,11). In the present series we observed no such relation, since in no case did the manual probe depth exceed 3 mm – the most frequent value being 1.5 mm.

According to our results, the dimensions of the DGU elements associated to tooth 21 with APE are a thick bone crest and connective tissue attachment, a long biological space, and a short distance from cementoenamel junction to bone crest.

Taking into account these three characteristics, it may be proposed that APE is characterized at DGU level by a long junctional epithelium, since the gingival sulcus appears normal and the biological length is prolonged, with a short connective tissue attachment - since the distance from crest to cementoenamel junction is short.

A gummy smile is accepted in the literature as one of the manifestations most suggestive of APE (12,13). Our own results support this notion. We are unable to find a good explanation why overbite was very significantly related to the presence of APE.

When tooth 21 presented APE (overlap > 19%), the lengths of the clinical crowns of all the teeth in the upper anterior sextant were significantly less than in the rest of the series, while in contrast gingival width was greater– with the sole exception of the canines ( Table 1). These results suggest that APE usually affect to all the teeth of 2º sextant.

An evaluation was made of the possible presence in our series of the two morphological patterns of APE described in the literature: type 1, characterized by a long distance from cementoenamel junction to bone crest together with low discrepancy; and type 2, defined by a short distance from cementoenamel junction to crest together with important discrepancy (6,10). From the statistical point of view, our series of patients presents these two morphological patterns of APE. These results coincide with the hypothesis postulated by Coslet (6) and posteriorly evaluated by Amsterdam, who explained these two morphological situations in terms of eruption arrest in either the passive or active phase. These authors defined APE type 2 in terms of a bone crest-cementoenamel junction distance of under 1.5 mm, and related it to the typical morphology of the DGU in erupting teeth in children and adolescents (6). In our series the mean distance was found to be 1.23 and 2.53 mm for APE type 2 and 1, respectively ( Table 3).

Although these two morphological types are seen in clinical practice, it is difficult to establish whether they are effectively attributable to the two proposed mechanisms. Both Ten Cate and Korbendau suggested that supracrestal connective tissue attachment is configured early during tooth formation, and that these collagen fibers attached to the root surface organize and become functional when the tooth erupts (14,15). Therefore, the space between the bone crest and cementoenamel junction may already be determined from this pre-eruptive stage. Our own results support this idea. If the mechanism of eruption were the sole responsible factor, it would be logical to assume that with active tooth eruption failure, some patient in the series could present a zero or even negative distance, as a result of burying of the tooth within the alveolar bone.

However, these results may also be interpreted taking both hypotheses into account, i.e., on one hand connective tissue attachment may be predetermined from before eruption of the tooth, while on the other the actual eruption mechanism might condition the definitive dimension. Studies are needed to clarify the position of the bone crest during the eruption process and establish how biological width is determined.

We thus raise the hypothesis that APE implies relative failure to fully complete tooth eruption, conditioned by two types of mechanisms:

(a) Compromised free space or tooth eruption, proving insufficient for the crowns of the antagonist teeth to fully erupt to occlusion. Such space restrictions could be conditioned by the type of facial growth pattern involved, and which would ultimately regulate the vertical space relationship between the two basal maxillary and mandibular bones.

(b) The second mechanism would imply the dimensional characteristics of the periodontal tissues surrounding the tooth. Accordingly, a disproportionate dimension of these tissues with respect to tooth size or eruption capacity would complicate both passage of the tooth during the active eruption phase and tissue withdrawal during the passive phase of eruption.

It therefore seems inappropriate to use the term APE to indicate this anatomical situation when mechanisms as difficult to study as tooth eruption appear to be involved. Other more cautious terms may be proposed as alternatives, such as for example incomplete crown exposure or excessive gingival overlap.

## Conclusions

1. Based on the results of the present study, altered passive eruption (APE) can be defined as a variant of habitual periodontal morphology, characterized at upper central incisor level by gingival overlapping on the anatomical crown equivalent to > 19% of its height. From the clinical perspective, APE is associated to increased gingival band width and gingival exposure on smiling.

2. At the dentogingival junction (DGU), the presence of APE is associated to a thick bone crest and connective tissue attachment, with a long biological space.

3. Statistical analysis confirms the presence of two morphological patterns of APE, respectively characterized by a longer and shorter distance from cementoenamel junction to bone crest.
